# A framework for research linking weather, climate and COVID-19

**DOI:** 10.1038/s41467-020-19546-7

**Published:** 2020-11-12

**Authors:** Benjamin F. Zaitchik, Neville Sweijd, Joy Shumake-Guillemot, Andy Morse, Chris Gordon, Aileen Marty, Juli Trtanj, Juerg Luterbacher, Joel Botai, Swadhin Behera, Yonglong Lu, Jane Olwoch, Ken Takahashi, Jennifer D. Stowell, Xavier Rodó

**Affiliations:** 1 Department of Earth and Planetary Sciences, Johns Hopkins University, 3400N. Charles St., Baltimore, MD 21218 USA; 2 Alliance for Collaboration on Climate and Earth Systems Science (ACCESS) c/o Council for Scientific and Industrial Research (CSIR), 15 Lower Hope Road, Cape Town, 7700 South Africa; 3 WHO/WMO Climate and Health Joint Office, World Meteorological Organization, 7bis Avenue de la Paix, C.P. 2300, CH-1211 Geneva, Switzerland; 4 School of Environmental Sciences, University of Liverpool, Liverpool, L69 7BX UK; 5 CDKN CEL-Ghana and Institute for Environment and Sanitation Studies, College of Basic and Applied Sciences, University of Ghana, Legon, Accra Ghana; 6 Herbert Wertheim College of Medicine, 11200 SW 8th St, AHC2 675, Miami, FL 33199 USA; 7 Climate Program Office, National Oceanic and Atmospheric Administration, 1315 East-West Highway Suite 100, Silver Spring, MD 20910 USA; 8 Science and Innovation Department, World Meteorological Organization, 7bis Avenue de la Paix, C.P. 2300, CH-1211 Geneva, Switzerland; 9 South African Weather Service, 01 Ecopark Drive, Ecoglades Block B, Centurion, Pretoria, 0157 South Africa; 10 Application Laboratory, VAiG, JAMSTEC, Yokohama, Japan; 11 Key Laboratory of the Ministry of Education for Coastal Wetland Ecosystems, College of the Environment and Ecology, Xiamen University, 361102 Fujian, China; 12 SASSCAL Regional Secretariat, 28 Robert Mugabe Avenue, Windhoek, Namibia; 13 Servicio Nacional de Meteorología e Hidrología del Perú–SENAMHI, Jr. Cahuide 785, Jesús María, Lima, 15072 Peru; 14 Boston University, 715 Albany Street, The Talbot Building, T4W, Boston, MA 02118 USA; 15 ICREA and Climate and Health Program, ISGlobal, Doctor Aiguader 88, Barcelona, 08003 Barcelona, Spain

**Keywords:** Ecological epidemiology, SARS-CoV-2, Climate sciences, Infectious diseases

## Abstract

Early studies of weather, seasonality, and environmental influences on COVID-19 have yielded inconsistent and confusing results. To provide policy-makers and the public with meaningful and actionable environmentally-informed COVID-19 risk estimates, the research community must meet robust methodological and communication standards.

When COVID-19 began to spread, environmental scientists recognized that the world faced a dangerous upper respiratory viral disease that might exhibit sensitivity to seasonal weather conditions. Many of these scientists have sought to aid COVID-19 response by studying the potential to monitor, forecast, or project disease transmission rates or symptom severity as a function of climate zone, season, meteorological variability, air quality, and other environmental parameters^1–4^. The rapid pace of COVID-19 research has meant that studies on this topic appeared on pre-print servers and then on news and social media outlets faster than the information could be cross-checked and peer-reviewed. As many such studies accumulated, it became clear that reported evidence was often contradictory, and in some cases studies were being selected subjectively in a manner that seemed intended to support political agendas. Carlson et al.^5^ recently provided a cogent assessment of the policy-relevant challenges associated with studies that have attempted to quantify meteorological sensitivities of the virus and the disease. We appreciate this perspective. Here we argue that the research community must act to ensure that work on this topic meets its potential to contribute to pandemic understanding and response, and that fears of inappropriate data analysis or miscommunication do not dampen innovation or the effective use of research results.

Environmentally-informed disease risk monitoring and prediction has proven to be useful for numerous infectious diseases^6^, including viral upper respiratory infections^7^ like COVID-19. These disease risk forecasts have been applied to vaccination strategies^8^ and have demonstrated value for informing deployment of non-pharmaceutical preventative measures and treatments^9,10^. In the case of COVID-19, environmentally-informed analysis has already revealed associations between chronic air pollution exposure and the health impacts of the disease^11^. The veracity of forecasts of COVID-19 incidence based on seasonality or other meteorological factors, however, continues to be in dispute, and the creation of robust disease risk forecast systems has been limited by data challenges^12^ and the dominance of or interplay with other drivers and pressures in the early phases of a respiratory disease epidemic^13^. If and when more consistent and meaningful climatic and environmental sensitivities are identified, such disease forecasts have the potential to help policy makers and public health officials target interventions in a way that optimizes effectiveness while minimizing their social and economic burden. Seasonally-informed physical distancing, other personal protection policies, and climate-informed vaccination strategies, for example, could make meaningful contributions to COVID-19 control, if the evidence that underlies science-based recommendations is robust and credible.

To realize this potential, however, pitfalls of miscommunication, use of unsuitable data and methods, and misrepresentation of results must be carefully guarded against. The interdisciplinary research and operational forecasting communities can do this by adopting a set of good practices for publishing, publicizing, and operationalizing their work. Recognizing this need, the World Meteorological Organization (WMO) convened an international virtual symposium on Climatological, Meteorological and Environmental factors in the COVID-19 pandemic, held 4–6 August 2020. The symposium engaged over 400 participants from 72 countries to assess and review current understanding, forecasting, and communication challenges related to climatic, meteorological and environmental influences on SARS-CoV-2 and COVID-19. The outcomes statement of this symposium^14^ offers pragmatic recommendations to the research and practitioner communities when pursuing their research and preparing it for scientific review or public consumption. These recommendations complement those directed at policy makers^5^. To build on the symposium, the WMO Research Board has established a Task Team on COVID-19 and Climatic, Meteorological, and Environmental Factors that is tasked with producing regular scientific assessments of the literature and formalizing recommendations on good practice for studies and creation of operational disease risk forecasting products that address these issues. This internationally coordinated effort is intended to promote constructive interdisciplinary collaboration and communication among researchers and decision makers.

A particularly strong message emerged from the symposium is that “*studies should attempt to meet a minimum standard of good practice in data use and methods for integrated models, including: the need to account for relevant non-environmental predictors; to justify the data quality and relevance of the selected response variable; to be clear about the epidemic phase being tested (including time lags); to spatially and temporally align epidemiological and climate, meteorological and environmental data; and to distinguish between analyses that are suited to describe observed relationships and those that have been confirmed to provide skillful prediction and forecast*”^14^. Symposium participants also emphasized that predictive studies must address the strength of mechanistic and dynamical understanding that underpins their empirical results. Ensuring that published studies have met these good practices will help to prevent many of the more egregious cases of overinterpretation or misinterpretation like those seen in the early stages of the pandemic, and will enhance the research community’s progress towards reliable and actionable results.

Robust research design and execution must, however, be accompanied by a culture of responsible publication and communication of results (Fig. 1). The very energy and civic-mindedness that has led so many from disparate disciplines to engage in the challenge of environmentally-informed COVID-19 disease risk prediction, has, at times, led to premature sharing of preliminary and sometimes unjustified results. The current scientific culture of releasing time-sensitive results on journal pre-print servers prior to peer review has resulted in a relaxation of the traditional self-regulation that research communities place on dissemination of results. The appearance of these unvetted studies online is often interpreted by journalists and the lay public, which is eager for certainty, as implying that they bear verified results. This presents a challenge to the COVID-19 research community, where a tension exists between the urgency to produce and publicize conclusive knowledge with the time and care needed for its authentication. While rapid communication of results, facilitated by journal pre-print publication, can accelerate the acquisition of knowledge and reduce redundancy, therefore allowing for faster progress on a critical research topic, pre-review release of studies can also mislead the public in the short term and threaten scientific credibility with decision makers in the medium and long term.

**Fig. 1:**
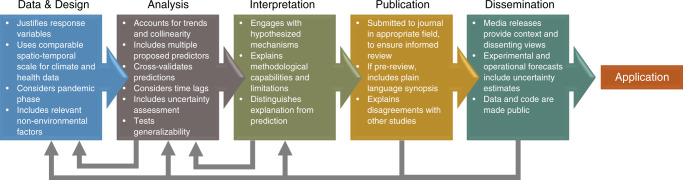
Template for generating reliable and actionable meteorologically and environmentally-informed COVID-19 risk analysis and prediction. Recognizing the rapid and high profile nature of COVID-19 research, investigators should apply careful checks throughout the Data and Design, Analysis, Interpretation, Publication, and Dissemination phases of their work. This includes iteration to ensure that dissemination of any policy relevant conclusions is grounded in study design and analysis, with results updated to reflect current understanding.

To balance opportunity with risk, journal and community pre-print servers should require a public synopsis, additional to the paper’s abstract, that states the study’s context in the literature—i.e., key contribution and agreement or disagreement with other studies, data sources, methodological approach and limitations, and proposed policy implications, if any. This would allow authors to clarify several common points of misinterpretation, such as whether a study is designed for prediction, how potentially confounding variables have been considered in the analysis, whether a model’s output is intended to be a specific forecast or a scenario-based projection, or if a statistically significant result (e.g., COVID-19 transmission sensitivity to temperature) is large or small relative to other risk factors.

In addition, researchers should embrace their role as representatives of the broader research community when communicating or operationalizing research results. This includes informing media outlets or policy makers of dissenting views and encouraging the presence of multiple voices in coverage of their work. It also includes clear presentation of uncertainties and alternative hypotheses in any publicly disseminated risk assessment or forecast—a practice that is well-established in climate and environmental change research. Such measures are good practice under any conditions, but researchers often leave the effort to others in the information chain, rather than viewing it as their own responsibility. In the rapidly evolving, multi-disciplinary, and potentially high stakes arena of COVID-19 forecasts, this is an avoided responsibility that the research community cannot afford. Through nimble but careful research and communication, it is possible to strike a balance between the rapid response research that the COVID-19 crisis demands and the need for careful review and communication of results likely to inform policy.
